# Battles against aberrant KEAP1-NRF2 signaling in lung cancer: intertwined metabolic and immune networks

**DOI:** 10.7150/thno.80184

**Published:** 2023-01-01

**Authors:** Ke Xu, Jie Ma, Sean R. R. Hall, Ren-Wang Peng, Haitang Yang, Feng Yao

**Affiliations:** 1 Department of Thoracic Surgery, Shanghai Chest Hospital, Shanghai Jiao Tong University, Shanghai, 200030, People's Republic of China.; 2 Department of Thoracic Surgery, Anhui Chest Hospital, Hefei, 230000, China; 3 Wyss Institute for Biologically Inspired Engineering, Harvard University; Boston, MA 02115, USA.; 4 Division of General Thoracic Surgery, Department of BioMedical Research (DBMR), Inselspital, Bern University Hospital, University of Bern; Bern, 3010, Switzerland.

**Keywords:** KEAP1-NRF2 signaling, non-small cell lung cancer, metabolic reprogramming, tumor immune microenvironment, bioinformatics, therapeutic vulnerabilities

## Abstract

The Kelch-like ECH-associated protein 1/nuclear factor erythroid-derived 2-like 2 (KEAP1/NRF2) pathway is well recognized as a key regulator of redox homeostasis, protecting cells from oxidative stress and xenobiotics under physiological circumstances. Cancer cells often hijack this pathway during initiation and progression, with aberrant KEAP1-NRF2 activity predominantly observed in non-small cell lung cancer (NSCLC), suggesting that cell/tissue-of-origin is likely to influence the genetic selection during malignant transformation. Hyperactivation of NRF2 confers a multi-faceted role, and recently, increasing evidence shows that a close interplay between metabolic reprogramming and tumor immunity remodelling contributes to its aggressiveness, treatment resistance (radio-/chemo-/immune-therapy) and susceptibility to metastases. Here, we discuss in detail the special metabolic and immune fitness enabled by KEAP1-NRF2 aberration in NSCLC. Furthermore, we summarize the similarities and differences in the dysregulated KEAP1-NRF2 pathway between two major histo-subtypes of NSCLC, provide mechanistic insights on the poor response to immunotherapy despite their high immunogenicity, and outline evolving strategies to treat this recalcitrant cancer subset. Finally, we integrate bioinformatic analysis of publicly available datasets to illustrate the new partners/effectors in NRF2-addicted cancer cells, which may provide new insights into context-directed treatment.

## Introduction

The Kelch-like ECH-associated protein 1 (KEAP1)-nuclear factor erythroid-derived 2-like 2 (NFE2L2) signaling pathway is well characterized by protecting cells from oxidative stress and xenobiotics. Under physiological conditions, KEAP1 binds to NRF2 and promotes its degradation through the ubiquitin-proteasome pathway, acting as an inhibitor of NRF2. Oxidative stress or electrophiles disrupt the KEAP1-NRF2 interaction, resulting in transient activation of NRF2 and subsequent translocation into the nucleus, where it binds to antioxidant response elements (ARE) in the genome. Consequently, transcription factor NRF2 activates several downstream genes including glutamate-cysteine ligase (GCL), heme oxygenase 1 (HO-1), NAD(P)H dehydrogenase (NQO1), glutathione S-transferase (GST) and thioredoxin reductase1 (TrxR1), involved in detoxification and antioxidant function.

NRF2 signaling is also frequently hijacked in various cancer types, most commonly in non-small cell lung cancer (NSCLC) 1, 2. In cancer, genetic alterations in *KEAP1* or *NFE2L2* (encoding NRF2) are the most common cause of NRF2 hyperactivation. The mutational landscape and the interaction between KEAP1 and NRF2 have been extensively covered in previous reviews 3-5. In addition, several other causes of NRF2 hyperactivation in cancer cells have also been described at genomic, transcriptional, and posttranslational levels. Specifically, CUL3 deletion or mutation results in the loss of KEAP1-dependent NRF2 ubiquitylation, causing aberrant NRF2 accumulations 6. Transcriptional changes, e.g., the loss of RBM47, are associated with KEAP1 mRNA stabilization, and the existence of alternatively spliced NRF2 variants also contributes to NRF2 augmentation 7, 8. Furthermore, sequestration of KEAP1 by NRF2-competitive-binding proteins including TRIM21, RPRD1A, and FAM129B was found in NRF2-addicted cancer models 9-11. Thus, it remains to explore the best way to define the NSCLC with high NRF2 addition. NRF2 score-based evaluation might be more feasible 12, 13.

The tumor micro-ecosystem contains cancer, stromal and immune cells, which corporately promote tumorigenesis and disease progression. Of the hallmarks of cancer, metabolism and immune evasion represent the most intertwined partners 14, 15. Accumulating evidence has revealed that aberrant KEAP1-NRF2 signaling influences both cancer cells and the cancer-associated microenvironment 16, 17, conferring specific metabolic vulnerabilities 18-20 and shaping a unique tumor immune microenvironment (TIME) 16, 21.

In this review, we focus on the role of the aberrant KEAP1-NRF2 signaling pathway in reprogramming tumor metabolism and modulating TIME in NSCLC. We also for the first time summarize the similarities and differences in the dysregulated KEAP1-NRF2 pathway between two major histo-subtypes of NSCLC. We combined literature review and mining of publicly available data to illustrate a more comprehensive landscape of dysregulated KEAP1-NRF2 pathway, which may contribute to a better understanding of the pathogenesis of this special disease and provide new directions for context-dependent treatment.

## KEAP1-NRF2 in different histo-subtypes of NSCLC: similarities and differences

**Figure 1:**
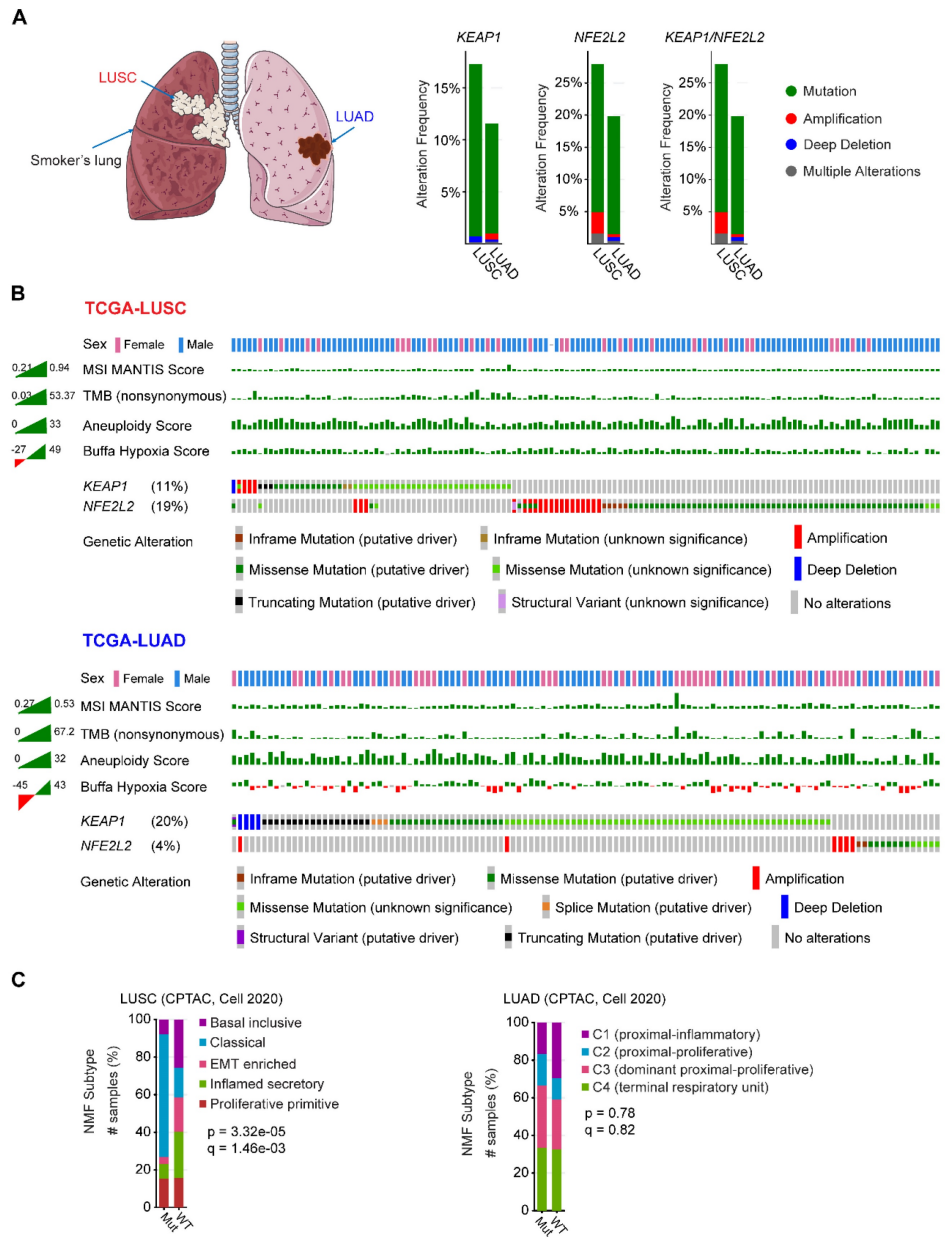
** Similarities and differences of KEAP1-NRF2 aberration in human non-small cell lung cancer.** A, Left panel: schematic model showing the common location of lung squamous cell carcinoma (LUSC; central; associated with a smoking history) and adenocarcinoma (LUAD; peripheral). Middle panel: key components of KEAP1-NRF2 pathway. Right panel: genetic alterations of *KEAP1, NFE2L2, CUL3* alone or in combination across The Cancer Genome Atlas (TCGA) LUSC and LUAD, respectively. Data were downloaded from the cBioPortal (https://www.cbioportal.org/). B, Oncoprint of genetic alterations of *KEAP1, NFE2L2, CUL3* across TCGA LUSC and LUAD. The corresponding patient sex, tumor mutational burden (TMB) and three curated signatures calculating the hypoxia score were also shown, which displayed a different distribution between LUSC and LUAD. Data were downloaded from the cBioPortal (https://www.cbioportal.org/). C, Comparing the transcriptional molecular features between mutated (Mut) (*KEAP1, NFE2L2, CUL3*) and wildtype (WT) in patient samples of LUSC and LUAD. Nonnegative matrix factorization (NMF) clustering was performed to identify the molecular subtypes. Data were downloaded from the cBioPortal (https://www.cbioportal.org/).

NSCLC contains two major histo-subtypes: lung squamous cell carcinoma (LUSC) and adenocarcinoma (LUAD), which originate in different cell types and are related to different risk factors (**Figure 1A**). The fact that genetic alterations in *KEAP1-NFE2L2* preferentially occur in human lung tumors suggests that the cell/tissue-of-origin is likely to influence the genetic selection that drives malignant transformation 22-24.

Mining publicly available genomic data from The Cancer Genome Atlas (TCGA) NSCLC cohort revealed that LUSC has a higher proportion of genetic alterations in *KEAP1/NFE2L2*, particularly *NFE2L2*, compared with its LUAD counterpart (**Figure 1A-B**). Furthermore, LUSC harboring *KEAP1-NFE2L2* alterations is observed with a higher frequency in male patients, and a differential distribution in the microsatellite instability (MSI), tumor mutational burden (TMB), aneuploidy, and notably, hypoxia scores could be observed between LUSC and LUAD (**Figure 1B**). Furthermore, non-negative matrix factorization-based clustering analysis of proteomic data also revealed markedly different molecular signatures between these two histo-subtypes (**Figure 1C**). These observations imply that *KEAP1-NFE2L2* alterations confer different biological behaviors in the two subtypes of NSCLC. Supporting this notion, recent multi-omics data of patient samples showed that unlike LUAD 25, *KEAP1* mutations did not result in significantly reduced protein expression in LUSC, suggesting the differences in NRF2 pathway dysregulation between NSCLC subtypes 26. Also, based on genetically engineered mouse models (GEEM), Li et al. showed convincing evidence that there is differential NRF2 and reactive oxygen species (ROS) levels between LUAD and LUSC 27. Strikingly, ROS functionally modulates LUAD to LUSC transdifferentiation to drive cancer progression and escape therapy. Accordingly, modulation of redox balance by overexpression of Nrf2 or treatment with ROS scavenger N-acetyl cysteine reduced the frequency of squamous tumors. Their data suggested that ROS function as a major driver of lung tumor differentiation between LUAD and LUSC. In addition, Keap1-deficient *Kras*^G12D^ mouse lung tumors arising from a bronchiolar cell-of-origin, lack pro-tumorigenic macrophages that are observed in tumors originating from alveolar cells 17, further reinforcing the notion that cells-of-origin influence the pathobiology of *KEAP1*-mutant NSCLC.

Although it remains to define the fundamental molecular underpinnings underlying the difference between the two NSCLC histo-subtypes with hyperactivated NRF2, the co-occurring mutations with *KEAP1/NFE2L2* genes might provide certain explanations. In LUAD, *KEAP1* mutation often co-exists with *STK11* and *KRAS*, whereas *NFE2L2* frequently co-occurs with *TP53* mutations in LUSC 28. Co-mutations with* KEAP1* and *STK11* configure a special metabolically and immunologically addicted phenotype in patients with *KRAS*-mutant LUAD 29. Besides, the *KEAP1* and *STK11* co-mutated LUAD also exhibits a ferroptosis-tolerate behavior, a newly discovered form of programmed cell death 30. In keeping with these data, clinical evidence shows that in *KEAP1*-mutant LUAD, co-mutated *PBRM1*, *SMARC4*, or *STK11* is associated with poor response to immunotherapy as compared with single-mutant or wild-type tumors 31. As to LUSC, the *KEAP1* and *TP53* co-mutations have been found in airway basal stem cells, promoting their renewal and expansion characteristics and conferring radiation resistance 32. While other co-mutations in LUSC are rarely mentioned. Thus, different co-mutated genes might also play a role in contributing to distinct biological behaviors between LUAD and LUSC.

Notably, the majority of preclinical mouse models focus predominantly on LUAD 16, 19, 20, 33, 34, but very few on LUSC 32. Although the regulation of oxidative stress response has been revealed in both subtypes, whether *KEAP1-NFE2L2* alterations hold equal weights in LUSC and LUAD, or whether the features of *KEAP1-NFEL2* alterations are governed by the cancer cell-of-origin warrants further investigations.

## Cancer Metabolic Reprogramming

Metabolic reprogramming is a hallmark of cancer, sustaining the survival and proliferation of cancer cells by regulating several key metabolic pathways such as glycolysis, glutaminolysis, and lipid metabolism to meet the elevated biosynthetic needs of tumors 35. Beyond its classical role in regulating oxidative stress, increasing evidence has revealed crosstalk between KEAP1-NRF2 signaling and tumor metabolic reprogramming 18-20, thereby creating targetable metabolic vulnerabilities. More recently, in a systematic study that aims to determine the redox vulnerability of KEAP1-NRF2-mutant NSCLC, CRISPR-Cas9-based screen for antioxidant enzymes led to the identification of multiple hits involved in the pentose phosphate pathway (PPP), the thioredoxin-dependent antioxidant system, and glutathione reductase, as well as mitochondrial superoxide dismutase 2 (SOD2) 36. This suggests that KEAP1-NRF2 signaling is related to a wide range of metabolic activities.

### Glucose metabolism

**Figure 2:**
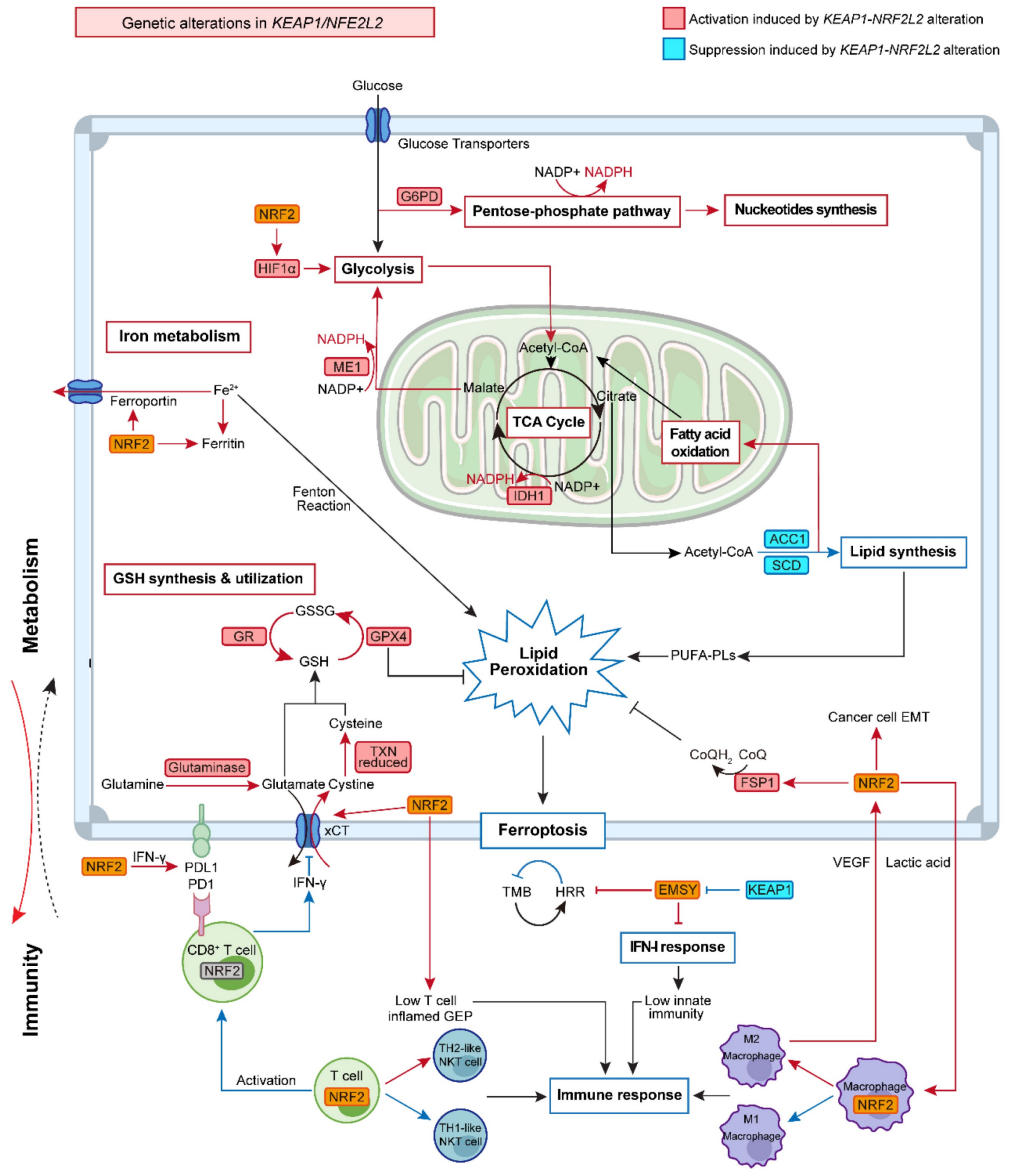
** Crosstalk between tumor metabolism and immunity in lung cancer with KEAP1-NRF2 aberration.** KEAP1-NRF2 aberration plays a pleiotropic role in tumor metabolism, which has a close interplay with tumor immunity

Aberrant glucose metabolism is a prominent type of metabolic reprogramming in cancer, which not only involves glycolysis that was preferentially used by tumors (Warburg effect) but also other pathways that require glucose, e.g., the PPP that generates pentose phosphates for ribonucleotide synthesis and NADPH for reducing equivalent 37. Recent studies indicated that KEAP1-NRF2 signaling drives glucose addiction in NSCLC cancer, and cancer cells with KEAP1 inactivation are more vulnerable to glucose deprivation (**Figure 2**) 18.

#### Glycolysis

**Figure 3:**
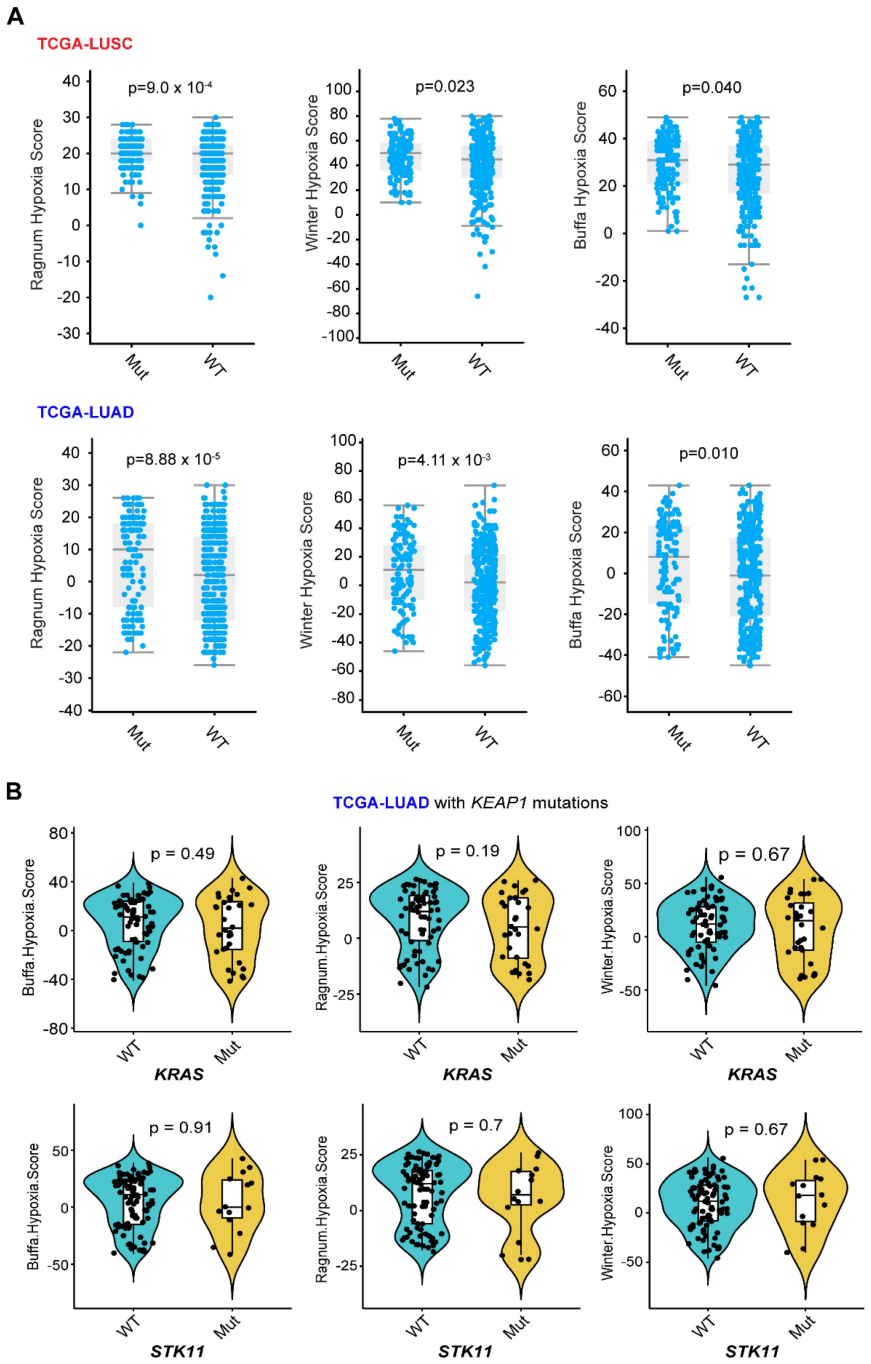
** Different hypoxia index between lung tumor with and without KEAP1-NRF2 aberration.** A, Three different curated signatures were used to compare the hypoxia score between mutated (Mut) (*KEAP1, NFE2L2, CUL3*) and wildtype (WT) in patient samples of TCGA LUSC (lung squamous cell carcinoma) and LUAD (lung adenocarcinoma). Data were downloaded from the cBioPortal (https://www.cbioportal.org/). Of note, the “subtle and marginal significance” between the comparative subgroups might be due to: 1) the presence of high NRF2 activity in the *KEAP1*-WT lung tumors, as NRF2 activity can be regulated at multiple levels including genetic, transcriptional, and post-transcriptional. As such, NRF2 score 12, 13 might better reflect the activity of NRF2 signaling. B, In LUAD, mutated *KEAP1* is commonly co-occurring with *KRAS* or *STK11* mutation. Different algorithms-based hypoxia scores were used to compare the difference between KRAS (upper panel) or STK11 (lower panel) wildtype (WT) and mutant (Mut) tumors under the context of KEAP1 mutation in LUAD. P-value was calculated by Wilcoxon Test.

The upregulation of glycolysis in cancer cells was related to hypoxia-inducible factor 1 α (HIF1α), followed by HIF1α-related glycolytic enzyme transcription 38. Analysis of publicly available data revealed that KEAP1-NRF2 alterations are overall related to a higher hypoxia score based on different algorithms (**Figure 3A**), and that the common genes co-occurring with *KEAP1* mutations in LUAD do not appear to affect their hypoxia status (**Figure 3B**). Of note, the above data-mining analysis revealed that mutated KEAP1-NRF2 in TCGA LUSC appears to be associated with a markedly higher hypoxia score (**Figure 1B**), while the biological explanation for this and the reason for the difference between LUSC and LUAD need further investigation.

The hyperactivation of the rate-limiting enzymes of the glycolytic pathway, e.g., hexokinase (HK) and pyruvate kinase (PK) was found in NRF2-addicted cancer 39. Furthermore, Zhang et al. demonstrated that NRF2 stimulates cancer cell progression by targeting HIF1α to enhance the expression of several key glycolytic genes including hexokinase 2 (HK2), phosphofructokinase-2/fructose-2,6-bisphosphatase 3 (PFKFB3), pyruvate kinase isozymes M2 (PKM2) and lactate dehydrogenase A (LDHA) 40. Similarly, Lee et al. showed that NRF2-silencing suppressed HIF1α accumulation and subsequently inhibited the expression of glycolysis-associated glucose transporter-1, HK2, pyruvate dehydrogenase kinase-1 (PDK1), and LDHA 41, suggesting the robust regulation of NRF2 on glycolysis is mediated by HIF1α. The interplay between HIF1α and NRF2 has also been revealed in other diseases 42, 43.

#### PPP and NADPH production

The PPP includes two distinct phases: the oxidative phase with NADPH production and the non-oxidative phase with 5-carbon sugars synthesis. Therefore, the PPP contributes to tumor proliferation not only by producing NADPH to buffer oxidative stress and prevent cells from death, but also by providing materials needed for nucleotide synthesis 44. The activation of these processes was also found to engage in NRF2-dependent tumor progression and metastasis.

Previous studies have reported that deregulation of the KEAP1-NRF2 signaling pathway promotes cellular proliferation and tumorigenesis *in vivo* by reprogramming glucose metabolism, with an altered metabolic profile characterized by increased glucose-derived carbon flux towards the PPP and tricarboxylic acid (TCA) cycle 45. NRF2 can directly regulate the transcription of various enzymes involved in the PPP. Specifically, expression products of genes involved in the oxidative phase (glucose-6-phosphate dehydrogenase [G6PD], 6-phosphogluconate dehydrogenase [6PGD]) and non-oxidative phase (transketolase [TKT], and transaldolase [TALDO1]) and *de novo* nucleotide synthesis (phosphoribosyl pyrophosphate amidotransferase [PPAT] and methylenetetrahydrofolate dehydrogenase 2 [MTHFD2]) were decreased by the NRF2 knockdown 45. In addition, sustained activation of NRF2 signaling in cancer cells was also found to indirectly enhance the PPP activation through the inhibition of microRNAs miR-1 and miR-206 expression. Specifically, sustained activation of NRF2 increased redox-sensitive histone deacetylase 4 (HDAC4) expression which attenuated miR-1 and miR-206 expression, leading to activation of PPP genes expression 46.

The regulation of PPP by NRF2 was also revealed by systematic investigations. Corroborating this, a metabolism-focused CRISPR screen demonstrated that G6PD was a top dependency in *KEAP1*-mutant lung tumors 47, which was supported by a more recent work by Jiang et al. 36. However, whether this dependency is different between *KEAP1*-mutant and -wildtype tumors remains to be defined. In an independent CRISPR metabolic screen, Zhao et al. revealed that mutational status in the KEAP1-NRF2 pathway influenced the sensitivity of oxidative PPP 48. Specifically, single knockout of oxidative PPP genes impacted the growth and survival of *KEAP1*-wildtype HeLa cells more dramatically than *KEAP1*-mutant lung A549 cells, which was presumably due to the fact that the increased glutathione (GSH) and higher expression of other NADPH-regenerating molecules contribute to the decreased dependence on oxidative PPP flux observed in cells expressing mutated KEAP1. The decreased importance of oxidative PPP for NADPH production and less essentiality of oxidative PPP genes in *KEAP1*-mutant cancer seem to be at odds with previous work highlighting a dependence on PPP by *KEAP1*-mutant tumors 36, 45, 47. The observed differences might be due to transient metabolic reprogramming by CRISPR knockout.

In addition to producing NADPH through modulating the PPP pathway, NRF2 also promotes NADPH production by directly regulating several other metabolic pathways. Specifically, isocitrate dehydrogenase (IDH1 and IDH2) and malic enzyme (ME1, ME2, and ME3), two main enzymes that catalyze isocitrate decarboxylation and the oxidative decarboxylation of malate, respectively, are accompanied by NADPH production 49. It has been demonstrated that cytosolic ME1 together with mitochondrial IDH2 supports tumor growth and metastasis 50. Interestingly, NRF2 was found to increase NADPH production by regulating the transcription of ME1 and IDH1 45.

Taken together, glucose metabolic reprogramming mediated by NRF2 plays a critical role in cancer cell survival and progression not only by providing materials and energy needed for rapid cell proliferation but also by producing NADPH for antioxidant synthesis to overcome oxidant stress.

### Amino Acid

Altered amino acid metabolism is another metabolic alteration that occurs in cancer cells for sustaining their uncontrolled proliferation. In addition to directly acting as substrates for protein synthesis, they can also take part in the process of energy generation, nucleoside synthesis, and cellular redox homeostasis maintenance 51. Recent studies also established a link between altered KEAP1-NRF2 signaling and amino acid metabolism (**Figure 2**).

#### GSH synthesis and utilization

GSH, one of the most powerful ROS scavengers, plays a critical role in tumor progression and metastasis and has been confirmed to be modulated by downstream genes of NRF2. The synthesis of GSH requires cysteine, which is converted from cystine uptaken via system Xc^-^ 52. xCT, a subunit protein of Xc^-^ encoded by *SLC7A11*, is reported to be regulated by NRF2 53. Furthermore, NRF2 also participates in the reduction of cystine to cysteine through transcriptional regulation of thioredoxin (TXN) and thioredoxin reductase 1 (TXNRD1) 54, 55. After providing sufficient intracellular cysteine, GSH is then synthesized by the consecutive reactions of the two major enzymes including glutamate-cysteine ligase (GCL, a heterodimeric enzyme comprised of GCL catalytic subunit (GCLC) and GCL modifier subunit (GCLM)) and GSH synthetase (GSS) 56. In addition, NRF2 is also involved in GSH utilization by upregulating enzymes including glutathione reductase (GR), glutathione peroxidase, and glutathione S transferase.

In NSCLC, the upregulation of GSH resulting from constitutive activation of NRF2 contributes to stronger resistance to radiotherapy 32 and chemotherapy 34. Thus, targeting NRF2/GSH activity may represent a promising strategy to avoid chemoradiotherapy resistance, which has been recently validated 57.

Taken together, the overproduction of GSH regulated by NRF2 inhibits cellular damage by counteracting ROS which maintains redox homeostasis and decreases oxidative damage, contributing to cancer cell survival.

#### Glutamate

Multi-omics analysis integrating whole-exome sequencing, transcriptomic and metabolic profiling robustly demonstrated that glutamate excretion, cystine uptake, and GSH synthesis represent reproducible features of NRF2-addicted lung cancer cells 58. Glutamate not only participates in GSH synthesis catalyzed by GCLC and GCLM, but is also exported by xCT coupled with cystine uptake 59. Mechanistically, NRF2 modulates glutamine metabolism by upregulating glutaminase, which catalyzes glutamine to glutamate 60. Accordingly, *NRF2* knockdown decreased the incorporation of glutamate into GSH, indicating that glutamate production mediated by NRF2 could support GSH synthesis 45. A combination of CRISPR-Cas9-based genetic screening and metabolomic profiling by Romero et al. showed that *KEAP1/NFE2L2*-mutant lung cancer relies on increased glutaminolysis, and pharmacological inhibition of glutaminase exerts therapeutic efficacy 19. LeBoeuf et al. demonstrated that NRF2-mediated secretion of glutamate via activation of xCT suppressed non-essential amino acids (NEAAs) synthesis, which increases the dependency of cancer cells on exogenous NEAAs 61. Thus, restricting exogenous sources of NEAAs combined with glutaminase inhibition may represent a novel therapeutic strategy in lung tumors with alterations in the KEAP1-NRF2 pathway. Glutamine metabolism mediated by NRF2 activation also contributes to chemoresistance by restraining the assembly of stress granules, and glutaminase inhibitors can play a role in sensitizing cancer cells to chemotherapy 60. Together, the heavy reliance of NRF2-addicted cancers on glutamate metabolism may present an opportunity for therapeutic targeting in lung cancer.

#### Tryptophan

In addition to changes in amino acids engaged in cellular redox homeostasis maintenance, NRF2 activation also alters other forms of amino acid metabolism in cancer cells. Tryptophan (Trp) metabolism, a central hub involved in the regulation of immunological and neural functional processes, has been confirmed to be associated with tumor malignancy and immune evasion 62. Fahrmann et al. confirmed that aberrant NRF2 activation in LUAD alters the Trp metabolism through the kynurenine pathway, contributing to a tumor-promoting, immunosuppressed microenvironment 63. Specifically, NRF2 upregulates tryptophan-kynurenine enzyme kynureninase (KYNU) and elicits an immunosuppressive microenvironment by efficient activation of T-regulatory cells and increased expression of programmed cell death protein-1 (PD-1) and programmed cell death ligand-1 (PD-L1), eventually leading to poorer survival. This study not only indicates a novel mechanism of NRF2 in Trp metabolism modulation but also provides evidence for the interaction between metabolic reprogramming and immunity remodelling in NRF2-addicted cancers, which will be discussed below in more detail.

### Lipid metabolism, iron metabolism and ferroptosis

Emerging evidence highlights the importance of lipid metabolism, which provides energy, signaling molecules, and source material for cell membrane synthesis, as a key regulator in the development of NSCLC 64. In the glucose-deficient tumor microenvironment (TME), lipid oxidation becomes the main route to generate NADH and FADH2 for ATP production 64, 65. In the lung, NRF2 regulates several lipases that are involved in degrading triglycerides and phospholipids, providing an important source of lipids 66. Additionally, previous studies confirmed that NRF2 plays a regulatory role in lipid synthesis by inhibiting two key enzymes, namely, stearoul CoA desaturate 1 (SCD1) and activate acetyl-CoA carboxylase 1 (ACC1). The downregulation of SCD1 and ACC1 results in higher fatty acid oxidation (FAO), which accelerates the oxidation of both long-chain and short-chain fatty acids within the mitochondria 67, 68. Other genes that regulate FAO include nuclear receptor retinoid X receptor alpha (RXRa), peroxisome proliferator-activated receptor-gamma (PPARg), and peroxisome proliferator-activated receptor delta (PPARδ) were also confirmed to be targeted by NRF2 69. These lines of evidence suggest a close relationship between NRF2 and lipid oxidation.

Interestingly, SCD1-catalyzed monounsaturated fatty acids (MUFAs) can replace polyunsaturated fatty acids (PUFAs) in the lipid membrane and decrease the accumulation of lipid peroxides, thus protecting cells against a form of regulated cell death termed ferroptosis that is driven by iron-dependent lipid peroxidation 70, 71. Disorder of iron metabolism induces an increased level of ferrous iron that interacts with hydrogen peroxide to form an excessive accumulation of reactive species via the Fenton reaction. The excess reactive species will then interact with PUFAs in the cytomembrane to generate excessive lipid peroxides. Previous studies have also demonstrated the critical role of NRF2 in modulating cellular iron metabolism by regulating genes involved in heme synthesis and hemoglobin catabolism 72, 73. As such, it is not surprising that the KEAP1-NRF2 pathway is involved in affecting this novel cell death cascade.

NRF2 not only upregulates the iron storage protein ferritin (FTL1, FTH1) to decrease labile iron levels but also modulates ferroportin, a transmembrane protein that exports iron out of the cell, which in turn diminishes the accumulation of free iron inside of the cell 60, 61. Sun et al. found that NRF2 nuclear accumulation activated the transcription of ferritin heavy chain-1 in hepatocellular carcinoma cells 74. Moreover, the cystine-glutamate transporter system xCT and glutathione peroxidase 4 (GPX4), the two main regulators in the ferroptosis process 71, were also found to be the downstream targets of NRF2 75. In agreement, recent work found that cancer cells were able to evade ferroptosis caused by xCT inhibition or GPX4 inhibition via activation of the NRF2-ARE pathway, and the inhibition of NRF2 can reverse the resistance to ferroptotic cell death 13, 76, reinforcing the notion that NRF2 plays a critical role in mediating the resistance to GPX4-dependent ferroptosis.

Recent evidence demonstrates the presence of a GPX4-independent ferroptosis network in cancer, e.g., ferroptosis suppressor protein 1 (FSP1, also known as AIFM2) 77. Intriguingly, FSP1 was identified as a novel transcriptional target of NRF2 57. The ubiquinone (CoQ)-FSP1 axis mediates ferroptosis- and radiation- resistance specifically in lung cancer cells harboring *KEAP1* mutations. Pharmacological inhibition of the CoQ-FSP1 axis re-sensitizes KEAP1-deficient lung cancer tumors, which are inherently resistant to radiotherapy, in part, by inducing ferroptosis. This study not only establishes CoQ-FSP1 as a new downstream effector of the KEAP1-NRF2 pathway but also provides an additional therapeutic target for treating *KEAP1*-mutant lung cancer 57.

In summary, NRF2 is an important transcriptional regulator of anti-ferroptosis genes which prevent lipid peroxidation and the accumulation of free iron. Meanwhile, this regulation also creates new therapeutic vulnerabilities.

## TIME

### The interplay between tumor metabolism and TIME under NRF2 addiction

**Figure 4:**
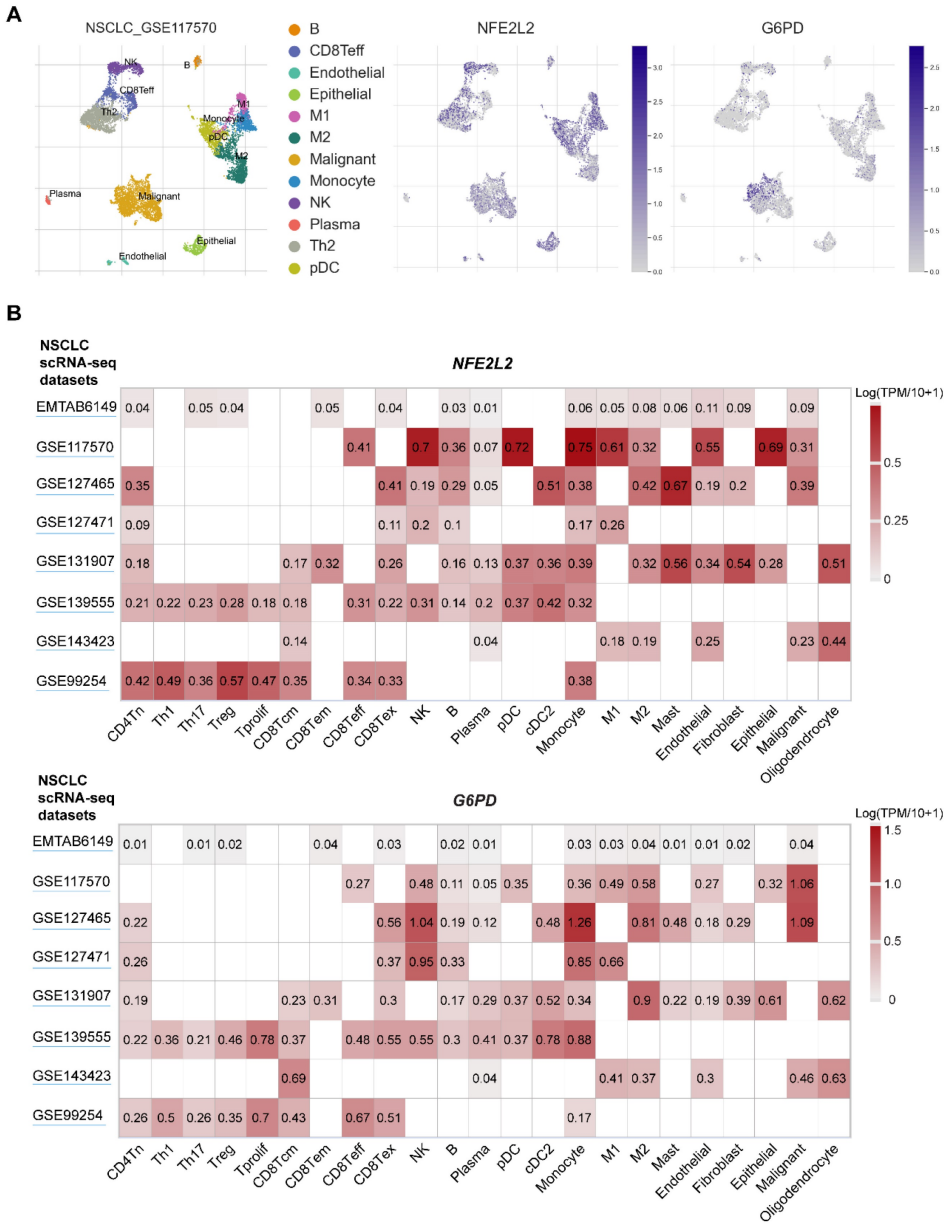
** scRNA-seq analysis of NRF2 expression across immune cell types within human NSCLC.** A, UMAP plots showing the unsupervised clustering of tumor and immune cells from one non-small cell lung cancer (NSCLC) patient cohort. The middle and right panels showing the expression of *NFE2L2* and *G6PD* (one key target gene of *NFE2L2*) across single cells. Single-cell RNA-seq (scRNA-seq) data were downloaded from the TISCH portal (Tumor Immune Single-cell Hub; http://tisch.comp-genomics.org/home/). B, Heatmap plots showing the distribution of *NFE2L2* and *G6PD* expression across different cell types in NSCLC.

Since genetic mutations are the driving force behind tumor phenotype, e.g., metabolic reprogramming enabled by mutated KEAP1, to support rapid growth and proliferation, thus it is rational to assume that KEAP1-NRF2 mutations in the cancer cell-of-origin promote a metabolic profile that shapes the TIME. As a return, the TIME signals back to the cancer cell, generating a feedback loop such that tumors and TIME co-evolve during progression. Supporting this notion, recent clinical studies demonstrate that the presence of NRF2 activation or *KEAP1* mutation in lung cancer is highly predictive of the unresponsiveness to immunotherapy 31, 78. These observations suggest that lung tumors with dysregulation of NRF2 signaling may be associated with a disordered TIME and a heavily compromised anti-tumor immune response. Mining publicly available single-cell RNA-sequencing (scRNA-seq) data in NSCLC reveals a wide distribution of *NFE2L2* and* G6PD* (a key downstream effector of NRF2) 45, 47 expression across cancer cells, as well as diverse immune cell types, particularly within the myeloid lineage (**Figure 4**).

Indeed, emerging evidence highlights that tumor-derived metabolites can modulate the surrounding TIME, which is particularly for glutamine metabolism, a key metabolic molecule engaged in the hyperactive NRF2 signaling 14, 29, 79, 80. In lung cancer, blocking the metabolism of glutamine to glutamate has pleiotropic effects on the immune system, such as reactivating CD8^+^T cells 29, enhancing PD-L1 expression 79, and decreasing myeloid-derived suppressor cells (MDSCs) 80. Of particular interest, glutamine blockade was shown to generate divergent effects on tumors and anti-tumor immune cells 14. Specifically, limiting glutamine uptake suppresses oxidative and glycolytic metabolism in cancer cells, whereas this markedly enhances oxidative metabolism and promotes a long-lived, highly activated phenotype in effector T cells 14. The differential reliance on nutrients was further supported by a recent study demonstrating that cancer cells depend on glutamine, whereas immune cells preferentially uptake glucose 81. As a result, blockage of glutamine utilization using glutamine transport inhibitors shows promise to overcome immunotherapy resistance 14, 29, 79, 80. Besides, some other forms of metabolism, e.g., ATP/adenosine regulated by ectonucleotidase CD39/CD73 82, fatty acid 83, and tryptophan 63 also play a critical role in modulating TIME. Together, metabolic reprogramming elicited by hyperactive NRF2 signaling can profoundly affect the surrounding TIME.

### Tumor immunogenicity

#### TMB/MSI

**Figure 5:**
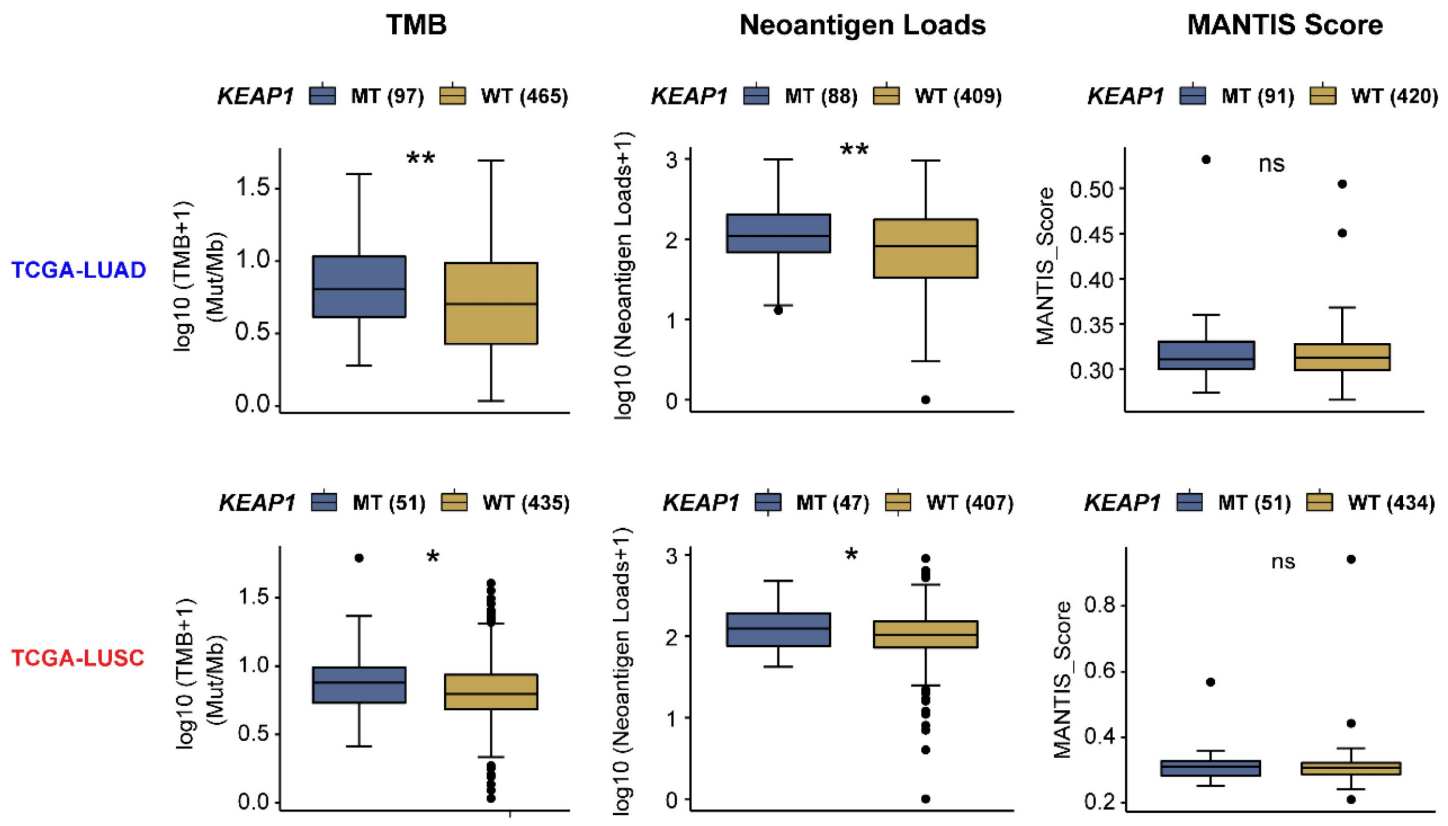
** The association of tumor immunogenicity with the mutant status of the KEAP1-NRF2 pathway in lung cancer.** Barplots showing the difference in tumor immunogenicity between *KEAP1*-mutant (mut) and -wildtype (WT) lung squamous cell carcinoma (LUSC) and adenocarcinoma (LUAD) samples of the TCGA cohort. Data were downloaded from the CAMOIP (Comprehensive Analysis on Multi-Omics of Immunotherapy in Pan-cancer) portal (https://www.camoip.net/).

Certain cancer cell-autonomous features of lung tumors, such as TMB, MSI, or neoantigen load, have been well-established to reflect host immunogenicity, and predict a good response to immunotherapy in patients with high expression of these features 84. Intriguingly, mining the TCGA NSCLC cohort, a significantly higher TMB and neoantigen load were observed in both *KEAP1*-mutant LUAD and LUSC (**Figure 5**); however, these features are not associated with a positive response to immunotherapy in this setting. In keeping with these data, clinical evidence shows that*KEAP1* is associated with poor response to immunotherapy despite the high TMB 31. Thus, the immunogenicity of *KEAP1*-mutant lung cancer cells is not likely to be the key determinant of the poor immunotherapy response. Conversely, reprogrammed TIME caused by *KEAP1* mutation may take the responsibility for this phenomenon.

Mechanistic insights on the observations that *KEAP1*-mutant lung cancer, despite the presence of high TMB, is associated with immunotherapy resistance remain largely unknown 31. Recently, Marzio et al. demonstrated that intact KEAP1 targets EMSY for ubiquitin-mediated degradation to regulate homologous recombination repair (HRR) and anti-tumor immunity 21. In contrast, loss of KEAP1 induces genomic instability due to the defective HRR, resulting in a high TMB through stabilizing EMSY. Critically, accumulated EMSY suppresses the type I interferon response and impairs innate immune signaling, thus promoting cancer immune evasion. Accordingly, activation of the type I interferon response in the TME using a STING agonist results in the engagement of innate and adaptive immune signaling and retards the growth of *KEAP1*-mutant lung tumors in mouse models. This study not only provides valuable insight into the role of *KEAP1* mutation in contributing to high TMB and immune escape but also important evidence for converting cold to hot tumors that are sensitive to immunotherapy. In agreement, Olagnier et al. declared that NRF2 activation represses STING expression by decreasing STING mRNA stability and impairs the responsiveness to STING agonists. Notably, the inhibitory effect of NRF2 on STING seems to be restricted in human cells rather than murine cells 85. Whether STING agonist represents an effective strategy to treat NRF2-addicted cells in humans warrants further exploration.

#### PD-L1

Immune checkpoint molecules regulate the balance between activation and suppression during the immune response, and their aberrant expression results in human cancers escaping immune surveillance. PD-1 and its ligand PD-L1 have been identified as immune checkpoint molecules and their interaction results in the immune suppression of T cells and immune evasion of cancer cells 86. Cancer cells of NSCLC show elevated levels of PD-L1 expression. Although the PD-1/PD-L1 treatment has significantly improved the survival of NSCLC patients, the overall response rate remains unsatisfactory 87.

Interestingly, a recent study identified a new mechanism for the KEAP1-NRF2 pathway in regulating immune checkpoint molecules such as PD-L1 88. The cullin (CUL) 3 is a component of Cullin-RING E3 ubiquitin ligase complex (CRLs) which are involved in protein ubiquitylation 89. The CUL3-speckle-type POZ protein (SPOP) E3 ligase complex has been confirmed to directly ubiquitinate PD-L1, leading to its degradation 90. Instead, the CUL3-KEAP1 complex was found to regulate the transcription of PD-L1 indirectly 88. The CUL3-KEAP1 complex stabilizes NRF2 protein, which leads to the upregulation of NRF2-mediated PD-L1 transcription after IFN-γ stimulation. The mechanism mentioned above may provide certain explanations for the findings that KEAP1 mutation or NRF2 activation is associated with resistance to immune checkpoint blockade therapy in lung cancer 31, 78, 91.

### T cells

T cells are at the core of adaptive immunity, and CD8+ T cell subsets form the backbone of effective immune checkpoint blockade (ICB) therapy. *Ex vivo* evidence based on the tumor-infiltrating T cells (TILs) sorted from tumor mass demonstrated a significant upregulation of NRF2 in TILs compared to matched uninvolved T cells. Interestingly, the authors observed that stimulating T cells by TCR activation significantly downregulated NRF2 expression and their target genes 92. Concordantly, IFN-γ production in NRF2-deficient T cells was dramatically enhanced 92. As mentioned above, KEAP1-NRF2 signaling plays a critical role in the anti-ferroptosis of cancer cells by regulating multiple target genes involved in cystine-GPX4-dependent and -independent pathways 57, 76. Interestingly, recent evidence demonstrated that under ICB treatment, CD8+ T cells-derived IFN-γ downregulates the two key subunits (SLC7A11, SLC3A2) of the glutamate-cystine antiporter xCT, thereby promoting tumor ferroptosis 93. This suggests that in the setting of NSCLC with aberrant KEAP1-NRF2 pathway, increased SLC7A11 expression might empower tumors to evade CD8-IFNγ-induced ferroptosis. In contrast to cancer cells, inhibiting ferroptosis in CD8+ T cells strongly restores their antitumor activity and enables greater antitumor efficacy in combination with ICBs 94.

Furthermore, *KEAP1* mutations are associated with a specific T cell-inflamed gene-expression profile (GEP), an emerging biomarker predicting antitumor responses of immunotherapy targeting the PD-1 axis in various tumor types 95. T cell-inflamed GEP includes 18 genes related to T cell-inflamed TIME 96. A recent pan-cancer analysis has revealed that tumors with *KEAP1* mutations have a poor response to ICB with a low level of T cell-inflamed GEP, and the immunomodulatory effects of *KEAP1* mutations appear to be specific to cancer types, especially in LUAD and LUSC 97. This may lead to tumor adaptation and subsequent immune suppression, allowing the survival and progression of lung cancer 97.

Despite this, there is a reason for caution. Staoh et al. found that NRF2 deficiency created an immunosuppressive microenvironment associated with a higher incidence of lung cancer metastasis following implantation of the mouse Lewis lung carcinoma cell line 98. In this study, MDSCs, which are immunosuppressive cells, were found to increase in the context of NRF2 deletion. However, an additional study by the same group showed that the increased level of MDSCs and decreased population of the CD8+ T-cells were also observed in the NRF2 wild-type mice bearing tumors 99. Another study also mentioned that the deletion of KEAP1 promoted tumor metastasis in patients with LUSC 32. The possible reason for this contradictory result is that NRF2 deficiency increased susceptibility of tumor initiations, whereas NRF2 activation promoted malignant progression at the later stage of cancer 99. These phenomena indicate a stage-related role of KEAP1-NRF2 signaling in immune modulation during tumor development, which suggests additional mechanisms requiring further investigation. Beyond that, co-mutations with *KEAP1* may also play a role. In human LUAD, *STK11* is one of the mutated genes that most co-occur with the *KEAP1* mutation. Previous evidence also established STK11 as a regulator of ROS 27. More recently, Sarah et al. investigated the metabolic and immune microenvironment of *KRAS*-mutant LUAD, based on *Keap1* and *STK11/Lkb1*-mutant mouse models. Surprisingly, they found that increased glutamate abundance was observed in the TME of* Lkb1*-mutant rather than *Keap1*-mutant LUAD, which was associated with CD8 T cell activation in response to the immune checkpoint inhibitors targeting PD-1-PD-L1 axis 29. Combination treatment with the glutaminase inhibitor CB-839 inhibited clonal expansion and activation of CD8 T cells 29, suggesting that glutaminase inhibition negatively impacts CD8 T cells activated by anti-PD-1 immunotherapy 100.

Apart from regulating conventional T cell proliferation and inflammation, the KEAP1-NRF2 pathway also participates in affecting the proliferation and function of innate T cells 101. Invariant natural killer T (NKT) cell, as the innate lineage of T cell, also plays an important role in immune responses against cancer 102. There are many subsets of NKT cells that play different immunomodulatory roles by secreting various cell-associated cytokines 103. For example, TH1-like NKT cell subsets have the potential to activate both tumor-specific T cells and natural killer (NK) cells to eliminate cancer cells, whereas TH2-/T regulatory-like NKT cell subsets exert immunosuppressive effects that facilitate tumor progression and immune escape 102. Along these lines, Pyaram et al. found an increased frequency of TH2-like NKT cells and a decreased frequency of TH1-like NKT cells in T cells with specific deletion of *KEAP1* 101, which creates an immunosuppressive microenvironment for cancer cells in this genetic context.

Altogether, the lung tumors with dysregulated KEAP1-NRF2 pathway appear to have profound effects on the T cells within the TIME, and disrupting this signaling is expected to promote the efficacy of ICB therapy.

### Macrophages

Among the immune cells within the TIME, lung macrophages represent the ones that are mostly exposed to a highly oxidative microenvironment, in that redox regulation is fundamental for phagocytic responses of macrophages 104. The MST1/2-NRF2 axis represents an important protective mechanism for macrophages during an antimicrobial response 105, and a link between NRF2/ERK signaling and ferroptosis is revealed in macrophages 106. Macrophages are comprised of two major subtypes: proinflammatory M1 macrophages phagocytose tumor cells, while anti-inflammatory M2 macrophages such as tumor‐associated macrophages (TAMs) stimulate tumor growth and invasion 107. ROS plays a crucial role in promoting macrophage polarization and modulates macrophage immunosuppressive phenotype 108. In the scRNA-seq dataset of NSCLC, *NFE2L2* and its key target gene *G6PD* are highly expressed, albeit heterogeneously, in M1 and M2 macrophages (**Figure 4**).

Kobayashi et al. reported that NRF2 plays an anti-inflammatory role by suppressing macrophage inflammatory response by inhibiting pro-inflammatory cytokines expression including IL-6 and IL-1β 109. Further study suggested that NRF2 contributes to the interaction between cancer cells and macrophages. Activation of NRF2 in cancer cells skews macrophage polarization towards an M2-like phenotype characterized by up-regulation of CD163 and Arg1, and down-regulation of IL-6 and IL-1β. The educated macrophages in turn activate NRF2 with VEGF expression which increases an epithelial-mesenchymal transition in cancer cells 110. However, direct evidence in the setting of *KEAP1*-mutant lung cancer is lacking, which warrants further studies.

### NK Cells

As mentioned above, previous studies have confirmed that KEAP1-NRF2 pathways influence T cells' proliferation and function. Since many signaling cascades that control NK cell effector function also participate in controlling T cell function 111, it can be reasonably hypothesized that the role of KEAP1-NRF2 pathways is also involved in NK cell regulation. NK cells carry out critical functions in innate immune surveillance in cancer 112. Deletion of both KEAP1 and PTEN was shown to result in decreasing number of NK cells in mice with lung cancer 16. Further, activation of NRF2 by the synthetic aromatic organic compound tert-butylhydroquinone (tBHQ) has been found to negatively affect the development and effector functions of NK cells 113.

Paradoxically, a recent study reports that the dysfunction of human NK cells in the TIME is due to suppressed glucose metabolism via lipid peroxidation-associated oxidative stress 114. Accordingly, expansion of NK cells with IL-21 treatment or an activator of NRF2 antioxidant pathway promotes Warburg-like metabolism with increased glycolysis and reduced oxidative phosphorylation, thereby leading to a markedly improved resistance to the oxidative stressed and nutrient-deprived TME, as well as enhanced *in vivo* antitumor responses. These findings suggest that activation of the NRF2 antioxidant pathway can restore the metabolic fitness and proper function of NK cells. Whether these observations exist *in situ* in the context of *KEAP1*-mutant NSCLC requires further investigations.

### Dendritic cells

Dendritic cells (DCs) represent a critical subset of innate immune cells that process and present tumor-derived antigens to prime T-cell immunity. tBHQ mentioned above was also confirmed to affect DCs through NRF2 activation 115. Specifically, tBHQ significantly attenuates IL-12 expression secreted by DCs, whereas the suppression can be prevented by genetic inhibition of NRF2 expression 115. In addition, the miR-200 family of microRNAs was also found to participate in the regulation of innate immune response related to DCs 116. MiR-200a can down-regulate DC maturation and prevents NRF2 ubiquitination caused by KEAP1 overexpression, which eventually reverses the inhibitory effect of DCs on tumors 117. Taken together, the overactivation of NRF2 contributes to cancer development partly through suppressing DCs function, although detailed mechanistic insights remain to be explored.

## Therapeutic target

Given the critical role of the KEAP1-NRF2 pathway in cancer, identifying novel compounds or repurposed drugs that can be used to target this pathway directly or indirectly is of great clinical interest for cancer treatment.

### Direct

#### Small molecule compounds

A natural compound, quassinoid brusatol that is extracted from *Brucea javanica*, was found to stimulate poly-ubiquitination of NRF2, leading to protein degradation 118. Of note, the inhibitory effect of brusatol on NRF2 is demonstrated to be irrespective of its classical repressor KEAP1. Another NRF2 inhibitor, halofuginone was shown to decrease Nrf2 at the protein level, through suppression prolyl-tRNA synthetase activity, and sensitize the chemotherapy on NRF2-addicted cancer cells 119. Akin to halofuginone, flavonoid luteolin was reported to accelerate the turnover of NRF2 mRNA, leading to a marked reduction of its mRNA and protein levels and enhanced sensitivity of lung cancer cells to chemotherapeutic agents 120. Furthermore, in a study screening ∼400,000 small molecules, ML385 was identified as a probe molecule that specifically binds to NRF2 and inhibits the expression of its downstream target gene 121. Specifically, ML385 binds to Neh1, the Cap 'N' Collar Basic Leucine Zipper (CNC-bZIP) domain of NRF2, and interferes with the binding of the V-Maf Avian Musculoaponeurotic Fibrosarcoma Oncogene Homologue G (MAFG)-NRF2 protein complex to regulatory DNA binding sequences 121.

#### Targeted protein degradation-PROTACs

NRF2 is typically degraded via the ubiquitination system. Targeted protein degradation is an emerging therapeutic approach to inhibit disease-causing proteins, such as transcription factors, that are historically challenging to target with conventional small molecules. Proteolysis targeting chimeras (PROTACs), which hijack E3 ligases and the ubiquitin-proteasome system (UPS) to selectively degrade the target proteins, represent a new class of promising therapeutic modalities 122 and has shown great promises to target previously undruggable targets. Beyond the KEAP1-Cullins3-RING-box protein 1 (KEAP1-Cul3-RBX1) axis, β-TRCP-S-phase kinase-associated protein 1-Cul1-RBX1 (SKP1-Cul1-RBX1) 123, and HMG-COA reductase degradation 1 homolog (HRD1) 124 have also been uncovered to control the ubiquitination of NRF2. Currently, multiple PROTACs-based or -alternative degrader approaches have been exploited in preclinical and clinical testing 122, 125, which may offer treatment potential in NRF2-addicted.

### Indirect

Directly targeting transcription factor NRF2 is still challenging, and efforts are mostly made on the collateral metabolic vulnerabilities rendered by NRF2, as depicted above that KEAP1-NRF2 signaling drives multiple metabolic dependences.

#### Dependent downstream effectors

Among the candidate targets, inhibition of glutaminase, the enzyme that catalyzes the conversion of glutamine to glutamate, represents the most promising one to treat NRF2-addicted NSCLC 18, 19, 59. Accordingly, the glutaminase inhibitor telaglenastat (CB-839) is being evaluated in phase II clinical trials, either in combination with chemo-immunotherapy or alone, in NSCLC patients whose tumors harbor *KEAP1* or *NFE2L2* mutations (NCT04265534 and NCT03872427). Liron et al. did chemical proteomics to identify druggable proteins that are selectively expressed in *KEAP1*-mutant NSCLC cells. Using this approach, the authors identified NR0B1, a transcriptional regulator that supports *KEAP1*-mutant NSCLC cells 8. Importantly, they identified the small compound BPK-29 that disrupts NR0B1 complexes and impairs the anchorage-independent growth of *KEAP1*-mutant cancer cells.

The dual mTORC1/2 inhibitor sapanisertib is being examined in the advanced setting in NSCLC (NCT02417701 and NCT04250545) based on preclinical evidence showing that *NFE2L2* mutations induce mTOR pathway dependency. Other targets including G6PD and endoplasmic reticulum-associated protein SLC33A1 20 have recently been validated as a new dependency specific in *KEAP1*-mutant NSCLC. More recently, CoQ-FSP1 axis was also shown to be specific to *KEAP1*-mutant NSCLC. Clinical evidence has shown that *KEAP1*-mutant lung tumors are highly refractory to radiotherapy 32. Inhibition of the CoQ biosynthesis enzyme COQ2 with 4-chlorobenzoic acid (4-CBA) strongly sensitized lung cancer cells to radiotherapy by inducing ferroptosis 57.

#### High-throughput screens

**Figure 6:**
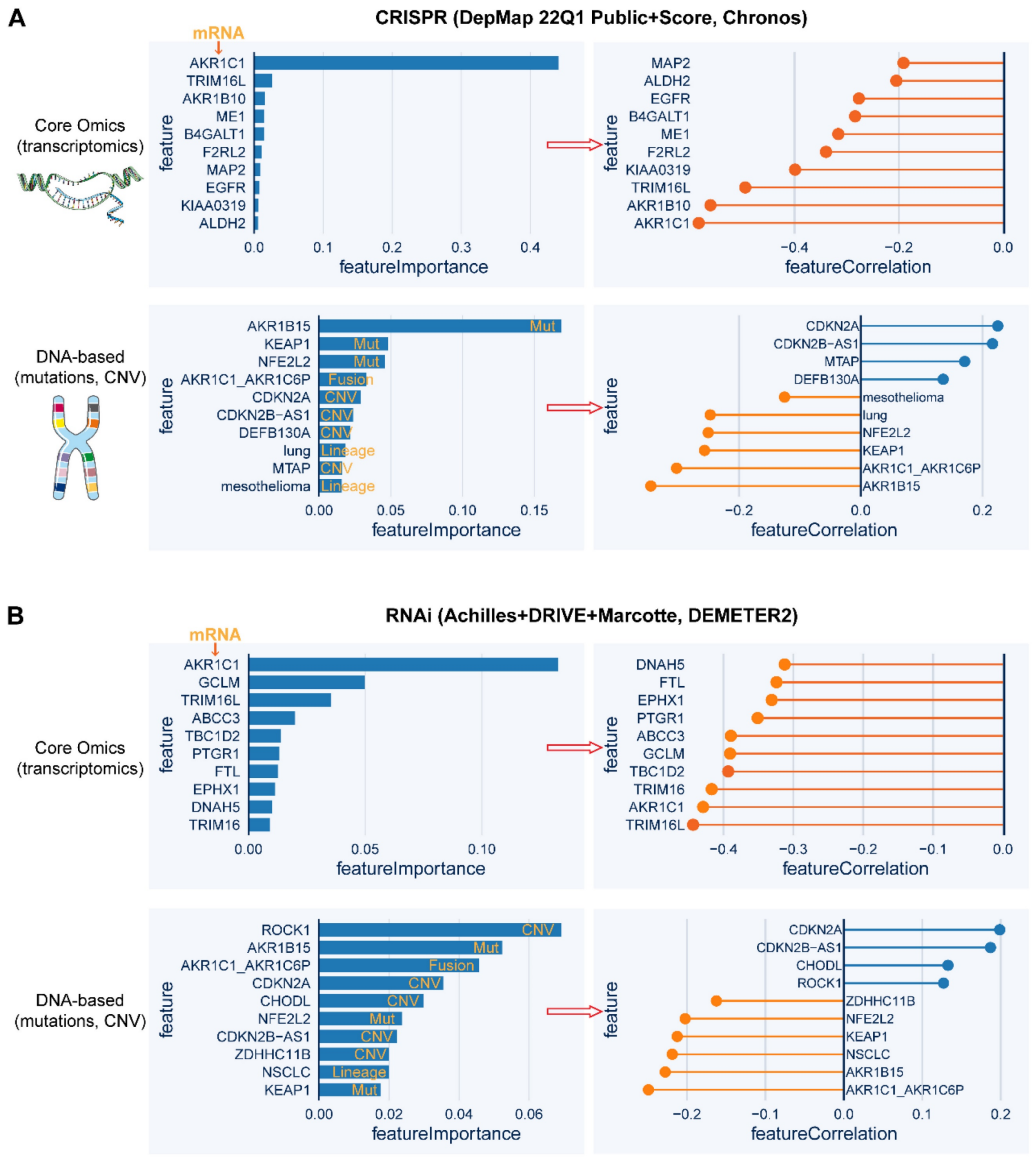
** Whole-genome screens reveal potential targeted dependency in *KEAP1*-mutant cancer cells.** A, B, Top 10 essential genes whose mRNA level (upper panel) or genetic mutation (lower panel) is correlated with the dependence score of *NFE2L2,* based on CRISPR (A)- and RNAi (B)-based whole-genome screen (https://depmap.org/portal/). In the left panels of A and B, the random forest machine learning strategy was used to identify the most important molecular features related to *NFE2L2* dependence. Right panels showing the Pearson correlation of the indicated genes with *NFE2L2* dependence score. The negative correlation indicates that genes whose higher expression or mutation signifies higher sensitivity to knockout or knockdown of *NFE2L2.*

Quantitative high-throughput screens can robustly facilitate the identification of genes whose expression or mutation is correlated with the degree of addiction to NRF2 function. Based on the publicly available datasets of CRISPR-Cas9 and RNA interference (RNAi) screening data, the top 10 genes whose mRNA level or genetic mutational status are tightly correlated (positively or negatively) with *NFE2L2* knockout or knockdown, respectively (**Figure 6A-B**). Specifically, in the CRISPR-Cas9 screen, *AKR1C1* expression represents the strongest feature that predicts the sensitivity to *NFE2L2* knockout, and strikingly, a genetic mutation in the AKR1B15 exhibits higher predictive power than *KEAP1* and *NFE2L2* mutations. The predictive ability of *AKR1C1* expression and mutational status of* KEAP1* and *NFE2L2* is also validated in the RNAi screen data. Interestingly, CDKN2A amplification is predicted to resist *NFE2L2* knockout or knockdown. These high-throughput analyses might provide a further understanding of the pathogenesis of NRF2-addictive cancer while also uncovering new therapeutic targets.

#### Mutation-directed new protein-protein interactions (PPI)

**Figure 7:**
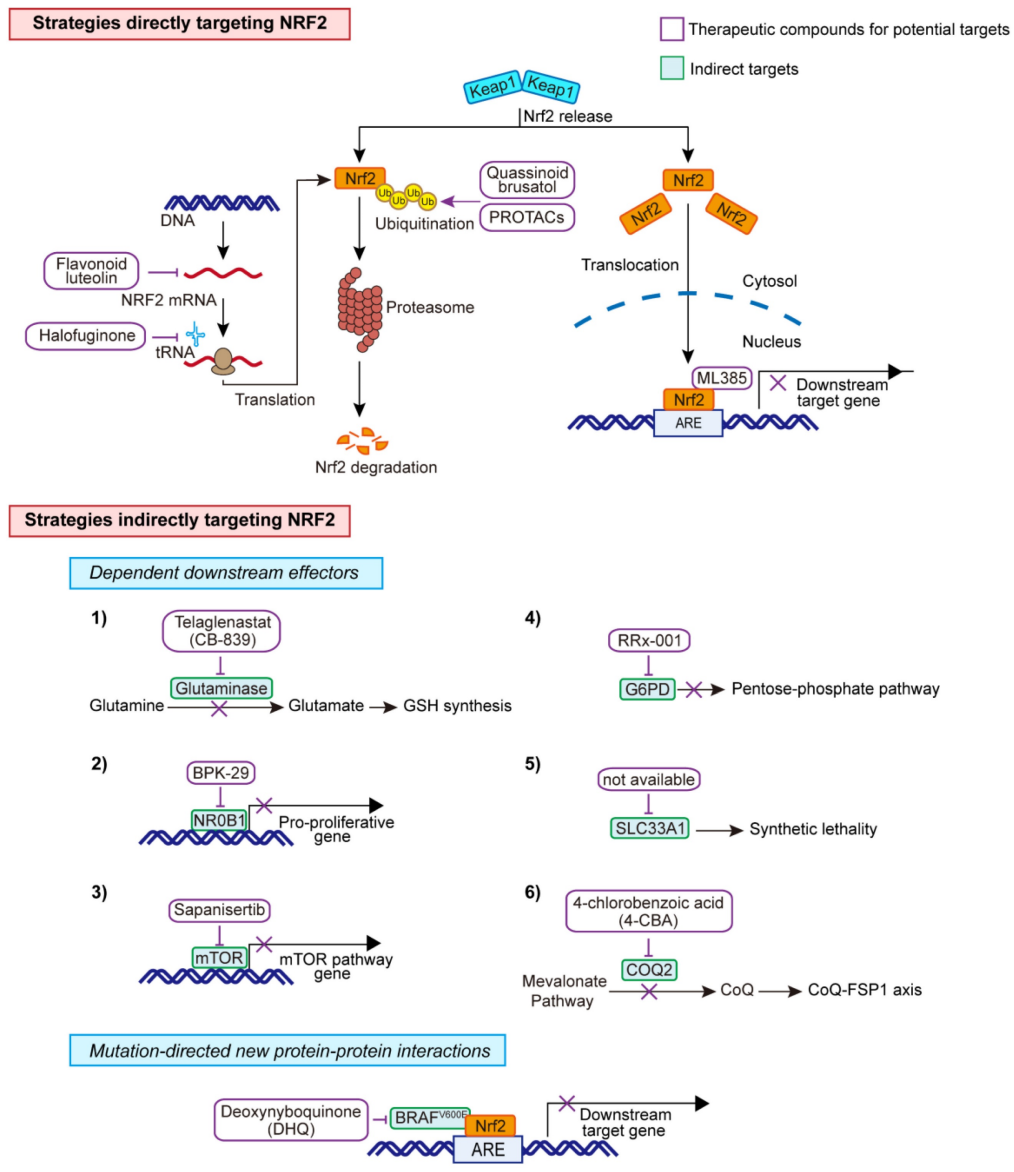
** Therapeutic strategies for *KEAP1*-mutant cancer cells.** Direct and indirect targets for the aberrant KEAP1-NRF2 pathway.

Mutations-driven loss or gain of PPIs illustrates another dimension of molecular underpinnings that enable tumorigenesis and cancer progression. In a recent study that aims to identify mutations-driven loss or gain of PPIs, the authors systematically performed the comparative analysis of wildtype and the paired mutant PPI profiles, uncovering an unappreciated function of mutated BRAF as a direct regulator of the KEAP1-mediated redox pathway through KEAP1-NRF2-ARE signaling, as overexpression of BRAF^V600E^, but not its wildtype counterpart, significantly stabilizes NRF2 protein and increases the transcriptional activity of ARE reporter 126. As a result, *BRAF^V600E^*-mutant cells are specifically vulnerable to quinone compounds such as deoxynyboquinone (DNQ). This example illustrates the potential of mutation-guided PPI to uncover oncogenic pathways as a promising strategy for pathobiological and therapeutic exploration, which is highly relevant for *KEAP1*-mutant lung tumors. The therapeutic strategies are summarized in **Figure 7**.

## Concluding remarks

Aberrant KEAP1-NRF2 signaling in NSCLC has both cancer cell-autonomous and non-autonomous effects, driving unique metabolic and immunological states, which suggests that combined targeting the metabolic vulnerabilities of cancer cells and disrupting their close interaction with the TIME would provide a more effective approach for this disease. Given the critical role of TIME in empowering NRF2-addicted NSCLC, new compounds or repurposed drugs that treat lung tumors with aberrant KEAP1-NRF2 signaling pathway should be tested in an intact immune system. Additionally, it is noteworthy that in preclinical mouse models, more attention should be paid to *KEAP1*-mutant LUSC, given that a higher incidence of mutations in KEAP1-NRF2 pathway occurs in LUSC, and more importantly, cell-of-origin in the lung might also matter in this genetic background. PROTACs and other alternative approaches show great promise in directly targeting the “undruggable” transcription factors, although their applications have not been achieved in the *KEAP1*-mutant setting. Other evolving strategies, e.g., mutations-driven loss or gain of PPIs, are also powerful to increase understanding of the KEAP1-NRF2 pathobiology, which is critical toward uncovering new therapeutic vulnerabilities of this daunting disease.

## Abbreviations

4-CBA: 4-chlorobenzoic acid
6PGD: 6-phosphogluconate dehydrogenase
ACC1: acetyl-CoA carboxylase 1
ARE: antioxidant response elements
CNC-bZIP: Cap 'N' Collar Basic Leucine Zipper
CoQ: ubiquinone
CRLs: cullin-RING E3 ubiquitin ligase complex
CUL: cullin
DCs: dendritic cells
DNQ: deoxynyboquinone
EpRE: electrophile-responsive element
FAO: fatty acid oxidation
FSP1: ferroptosis suppressor protein 1
G6PD: glucose-6-phosphate dehydrogenase
GCL: glutamate-cysteine ligase
GCL: glutamate-cysteine ligase
GCLC: GCL catalytic subunit
GCLM: GCL modifier subunit
GEEM: genetically engineered mouse models
GEP: gene-expression profile
GPX4: glutathione peroxidase 4
GR: glutathione reductase
GSH: glutathione
GSS: GSH synthetase
GST: glutathione S-transferase
HIF1α: hypoxia-inducible factor 1 α
HK: hexokinase
HO-1: heme oxygenase 1
HRD1: HMG-COA reductase degradation 1 homolog
HRR: homologous recombination repair
ICB: immune checkpoint blockade
IDH: isocitrate dehydrogenase
KEAP1: kelch-like ECH-associated protein 1
KEAP1-Cul3-RBX1: KEAP1-Cullins3-RING-box protein 1
LDHA: lactate dehydrogenase A
LUAD: lung adenocarcinoma
LUSC: lung squamous cell carcinoma
MAFG: V-Maf Avian Musculoaponeurotic Fibrosarcoma Oncogene Homologue G
MDSCs: myeloid-derived suppressor cells
ME: malic enzyme
MSI: microsatellite instability
MTHFD2: methylenetetrahydrofolate dehydrogenase 2
MUFAs: monounsaturated fatty acids
NEAAs: non-essential amino acids
NFE2L2: nuclear factor erythroid-derived 2-like 2
NK: natural killer
NKT: natural killer T
NQO1: NAD(P)H dehydrogenase
NSCLC: non-small cell lung cancer
PD-1: programmed cell death protein-1
PDK1: pyruvate dehydrogenase kinase-1
PD-L1: programmed cell death ligand-1
PFKFB3: phosphofructokinase-2/fructose-2,6-bisphosphatase 3
PK: pyruvate kinase
PKM2: pyruvate kinase isozymes M2
PPARg: peroxisome proliferator-activated receptor-gamma
PPARδ: peroxisome proliferator-activated receptor delta
PPAT: phosphoribosyl pyrophosphate amidotransferase
PPI: mutation-directed new protein-protein interactions
PPP: pentose phosphate pathway
PROTACs: proteolysis targeting chimeras
PUFAs: polyunsaturated fatty acids
RNAi: RNA interference
ROS: reactive oxygen species
RXRa: receptor retinoid X receptor alpha
SCD1: stearoul CoA desaturate 1
scRNA-seq: single-cell RNA-sequencing
SKP1-Cul1-RBX1: β-TRCP-S-phase kinase-associated protein 1-Cul1-RBX1
SOD2: mitochondrial superoxide dismutase 2
SPOP: speckle-type POZ protein
TALDO1: transaldolase
TAMs: tumor‐associated macrophages
tBHQ: tert-butylhydroquinone
TCA: tricarboxylic acid
TCGA: The Cancer Genome Atlas
TILs: infiltrating T cells
TIME: tumor immune microenvironment
TKT: transketolase
TME: tumor microenvironment
TMB: tumor mutational burden
TrxR1: thioredoxin reductase1
TXN: thioredoxin
UPS: ubiquitin-proteasome system
